# Evasion of Immunity to *Plasmodium falciparum*: Rosettes of Blood Group A Impair Recognition of PfEMP1

**DOI:** 10.1371/journal.pone.0145120

**Published:** 2015-12-29

**Authors:** Kirsten Moll, Mia Palmkvist, Junhong Ch'ng, Mpungu Steven Kiwuwa, Mats Wahlgren

**Affiliations:** 1 Department of Microbiology, Tumor and Cell Biology (MTC), Karolinska Institutet, Box 280, Nobels väg 16, SE-171 77 Stockholm, Sweden; 2 Department of Pediatrics, School of Medicine, Makerere University College of Health Sciences, Kampala, Uganda; 3 Department of Biochemistry, School of Biomedical Sciences, Makerere University College of Health Sciences, Kampala, Uganda; Universidade Federal de Minas Gerais, BRAZIL

## Abstract

The ABO blood group antigens are expressed on erythrocytes but also on endothelial cells, platelets and serum proteins. Notably, the ABO blood group of a malaria patient determines the development of the disease given that blood group O reduces the probability to succumb in severe malaria, compared to individuals of groups A, B or AB. *P*. *falciparum* rosetting and sequestration are mediated by PfEMP1, RIFIN and STEVOR, expressed at the surface of the parasitized red blood cell (pRBC). Antibodies to these antigens consequently modify the course of a malaria infection by preventing sequestration and promoting phagocytosis of pRBC. Here we have studied rosetting *P*. *falciparum* and present evidence of an immune evasion mechanism not previously recognized. We find the accessibility of antibodies to PfEMP1 at the surface of the pRBC to be reduced when *P*. *falciparum* forms rosettes in blood group A RBC, as compared to group O RBC. The pRBC surrounds itself with tightly bound normal RBC that makes PfEMP1 inaccessible to antibodies and clearance by the immune system. Accordingly, pRBC of *in vitro* cloned *P*. *falciparum* devoid of ABO blood group dependent rosetting were equally well detected by anti-PfEMP1 antibodies, independent of the blood group utilized for their propagation. The pathogenic mechanisms underlying the severe forms of malaria may in patients of blood group A depend on the ability of the parasite to mask PfEMP1 from antibody recognition, in so doing evading immune clearance.

## Introduction

The ABO blood group system was discovered about a century ago by the Austrian biologist and physician Karl Landsteiner. Several studies have reported associations between different infectious diseases and the distribution of the ABO blood groups [1–3] and a growing body of evidence suggests that *Plasmodium falciparum* has been a major selection force [4–6]. The prevalence of blood group O is high in malaria endemic areas and it matches the distribution of malaria. Further, a correlation between clinical severity of malaria and the ABO blood group of the patient is at hand since severe disease is overrepresented in individuals of non-O blood groups (A, B and AB, [7–12]). For rosette formation, a virulence phenomenon of *P*. *falciparum* where pRBC bind to uninfected RBC, the patient’s ABO blood group is also of importance since rosetting is more prominent in blood group A than in group O [11,13–15]. Further, children of group A have a higher probability to succumb in severe malaria as compared to children of blood group O [7,9,16,17]. Children of endemic areas are therefore selected by death in severe disease for blood group O, and red cell disorders such as sickle-cell trait and thalassemia, prior to fertile age, and *P*. *falciparum* malaria has in this manner driven the evolution of the ABO blood types [4], possibly due to rosetting [9,18].

The *Plasmodium* parasite has developed various ways to evade the host immune system during the erythrocytic part of the life cycle. The parasite escapes clearance by the spleen through sequestration of pRBC in the micro-vasculature because of binding to endothelial cells and to erythrocytes. Rosette formation has also been suggested to facilitate the invasion of merozoites into fresh erythrocytes by allowing “direct passage” from one RBC to the other i.e. minimal exposure to the host plasma [19–21]. The parasite may bury critical merozoite antigens from antibodies to allow the invasion process to proceed smoothly, but rosetting has so far not been found to enhance invasion *in vitro* [19–22]. Nevertheless, during schizogony rosetting is often followed by invasion of bound RBC, and the peripheral parasitemia, the level of rosetting and the rate of multiplication correlate positively to one another for individual isolates [19–21]. Rosetting is also more frequent with pRBC of children with severe versus uncomplicated malaria [23] but the associations that suggest rosetting to enhance the ability of the parasite to multiply within the human host have not been possible to confirm *in vitro* and rosetting parasites do not grow and multiply better than non-rosetting clones [24,25]. Recently, it has been suggested that pRBC which adhere in the placenta may escape antibody recognition given that non-immune IgM masks protective PfEMP1-epitopes on the pRBC surface [26]. Also rosetting has been suggested to protect the parasite against the host immune response by acting as a “cloaking device” hiding the pRBC from effector mechanisms of the immune system such as phagocytic cells [19,27].

This study pursues the hypothesis that rosetting contributes to immune evasion by hiding epitopes exposed on the pRBC surface when sequestered in the microvasculature. pRBC of parasite clones different in their preference for RBC of the ABO blood group system were evaluated for rosette formation and recognition of PfEMP1, among them the well characterized parasite clone FCR3S1.2. We here show that blood group A rosetting in FCR3S1.2, which depends to a large degree on a member of the RIFIN protein family (RIFIN-IT_5750_ [15]), blocks antibody recognition of PfEMP1-IT_var60_ [28], that mediates group O rosetting (Table 1). Other parasites were also found to vary in their rosetting preference for RBC of the ABO blood groups, as illustrated by large blood group A rosettes [13], and importantly, the diminished sensitivity of rosettes towards anti-PfEMP1 antibodies in blood group A as compared to O. Further, antibodies to PfEMP1 both disrupted rosettes formed by blood group O RBC and reacted with surface expressed PfEMP1, while the formation of antibody-resistant rosettes in blood group A significantly reduced antibody recognition. This suggests that antigens other than PfEMP1 such as RIFIN may contribute to group A rosetting, and therefore, large and tight rosettes in blood group other than group O may allow the parasite evade immune-responses to surface located antigens such as PfEMP1.

**Table 1:** Parasites used in the present study and their reactivity of the pRBC with different antibody preparations to surface expressed antigens (PfEMP1, RIFIN).

Parasite	Rosetting		IgG reactivity^1^			
		α-PfEMP1-IT-_var60_	α -RIF-3D7-_5750_	α -PfEMP1-IT-_var9_		α -PfEMP1- _varo_
FCR3S1.2 (IT)	≈85	+++^2^	++	-		-
R29 (IT)	≈70	-	-	+++		-
PAvarO	≈80	-	-	-		+++

1. Reactivity of pRBC in indirect immunofluorescence with Alexa 488 coupled anti goat IgG after incubation with 50μgs of goat IgG/ml purified from sera immunized with recombinant PfEMP1 of ITvar60 (FCR3S1.2), ITvar9 (R29), var1 (Palto AltovarO) or RIFIN-5750 (FCR3S1.2/3D7), see also Angeletti *et al* [51] and Goel et al [15].

2.— = no reactivity, +++ = strong reactivity.

## Results

### Blood group preference and size of rosettes

Four laboratory parasite clones (FCR3S1.2 [28], R29 [29], PAvarO [30], 3D7S8.4 [31]) and four patient isolates (UKS41, UKS55, UKS86, UKM104 [32]), all of a rosetting phenotype, were in parallel studied for formation of rosettes in RBC of blood group A and O and the size of rosettes was determined by microscopy.

**Fig 1:**
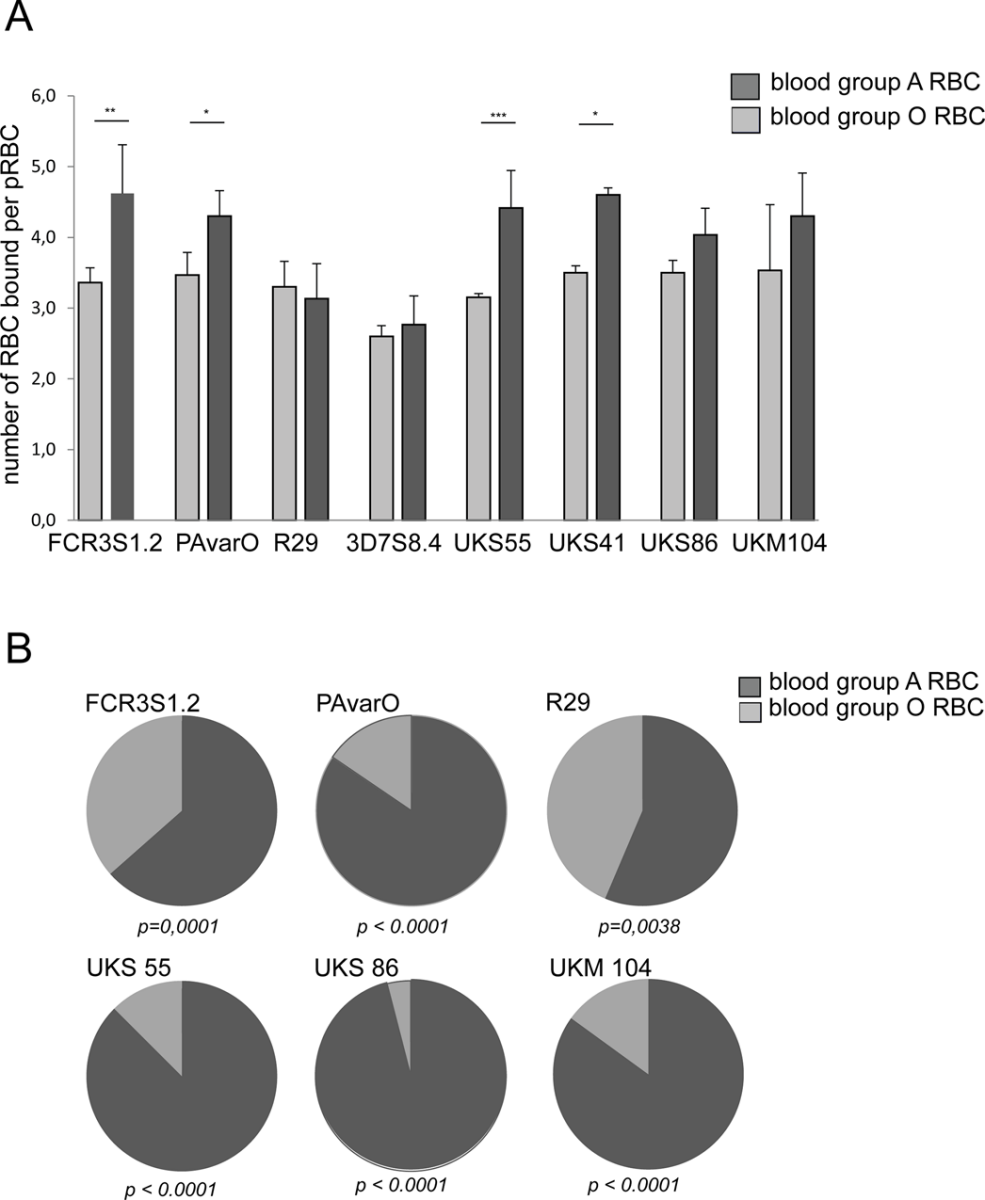
Rosette formation in blood group A versus O. **A:** Size of rosettes in blood group A versus O: Parasite laboratory clones FCR3S1.2, PAvarO, R29, 3D7S8.4 and the patient isolates UAS55, UKS41, UKS86, UKM104 were grown in parallel in blood group A or O RBC and the number of bound RBC per pRBC was determined at three independent occasions (five for FCR3S1.2); at each occasion 100 rosettes were investigated. FCR3S1.2 (p = 0.0044), PAvarO (p = 0.0404), UKS41 (p = 0.015) and UKS55 (p = 0.0001) formed significantly bigger rosettes in blood group A as compared to O (unpaired t test, two tailed). Bars represent mean of 3 (5) experiments plus SD. **B:** Preference of blood group A over O in the formation of rosettes: When assayed for preference of incorporation of RBC of blood group A or O at 3 independent occasions in duplicates (PKH labelling of either O or A RBC); in each experiment, 75 rosettes were investigated and the number of RBC of blood group A or O in each rosette was counted. All investigated parasite clones showed a significant preference to incorporate blood group A over O RBC in rosettes when exposed to a mixture of RBC of both blood groups; this preference was least profound in the clone R29 (p-values: FCR3S1.2: 0,0001; R29: 0,0038: PAvarO: < 0.0001; UKS55: < 0.0001; UKS86: < 0.0001; UKM104: < 0.0001; unpaired t test, two tailed p value). Graphs represent the mean of 3 experiments.

The laboratory clones FCR3S1.2, PAvarO as well as patient isolates UKS41 and UKS55 formed significantly bigger rosettes in blood group A as compared to group O RBC (FCR3S1.2 blood group A, 4.6 RBC/rosette, blood group O, 3.4 RBC/rosette, p = 0.0044; PAvarO, blood group A 4.3, blood group O 3.5 RBC/rosette, p = 0,0404; UKS41, blood group A, 4.4, blood group O, 3.2 RBC/rosette, p = 0,015; UKS55, blood group A, 4.6, blood group O, 3.5 RBC/rosette, p = 0,0001; unpaired t-test two tail), (Fig 1A). In contrast, rosettes formed by R29 (IT4 clone), 3D7S8.4 (3D7 clone), patient isolates UKS86 and UKM104 did not show a significant difference in size with RBC of the two blood groups (R29 blood group A, 3.1, blood group O, 3.3 RBC/rosette; 3D7S8.4, blood group A, 2.6, blood group O, 2.8 RBC/rosette; UKS86, blood group A, 4.2, blood group O, 3.4 RBC/rosette; UKM104, blood group A, 4.3, blood group O, 3.5 RBC/rosette; unpaired t- test two tail).

Preference of pRBC of different parasites for ABO blood group in rosette formation was also accessed by mixing MACS enriched pRBC propagated in blood group O RBC with RBC of blood groups A or O RBC pre-labeled with fluorescence dyes (PKH67 or PKH26). Equal numbers of labeled O and A RBC were added to pRBC and co-incubated at RT for 1 hour after which the composition of mixed rosettes was analyzed by microscopy. All investigated parasites showed a preference for incorporating blood group A over O RBC in rosettes, however, this tendency was less distinct for pRBC of the clone R29 as compared to the laboratory strains FCR3S1.2 and PAvarO (FCR3S1.2, blood group A, 64%, blood group O, 36%, p = 0,0001; PAvarO blood group A 85%, blood group O 15%, p< 0.0001; R29 blood group A, 56%, blood group O, 44%; p = 0,0038; UKS55 blood group A, 88%, blood group O 12%; p< 0.0001; UKS86 blood group A, 96%, blood group O, 4%; p< 0.0001; UKM104 blood group A, 85%, blood group O, 15%, p< 0.0001; unpaired t test, two tailed p value) (Fig 1B). Rosettes of 3D7S8.4 were not possible to reconstitute after MACS enrichment with the protocol used indicating a very weak rosetting-interaction of this parasite; pRBC of UKS41 were not efficiently enriched with MACS purification. Taken together with the above, we conclude that all investigated parasite clones/isolates have a preference in rosette-formation for A RBC vs. group O RBC but only a subset of parasites also form larger rosettes with blood group A RBC.

### Antibody disruption of blood group A rosettes is impaired

**Fig 2:**
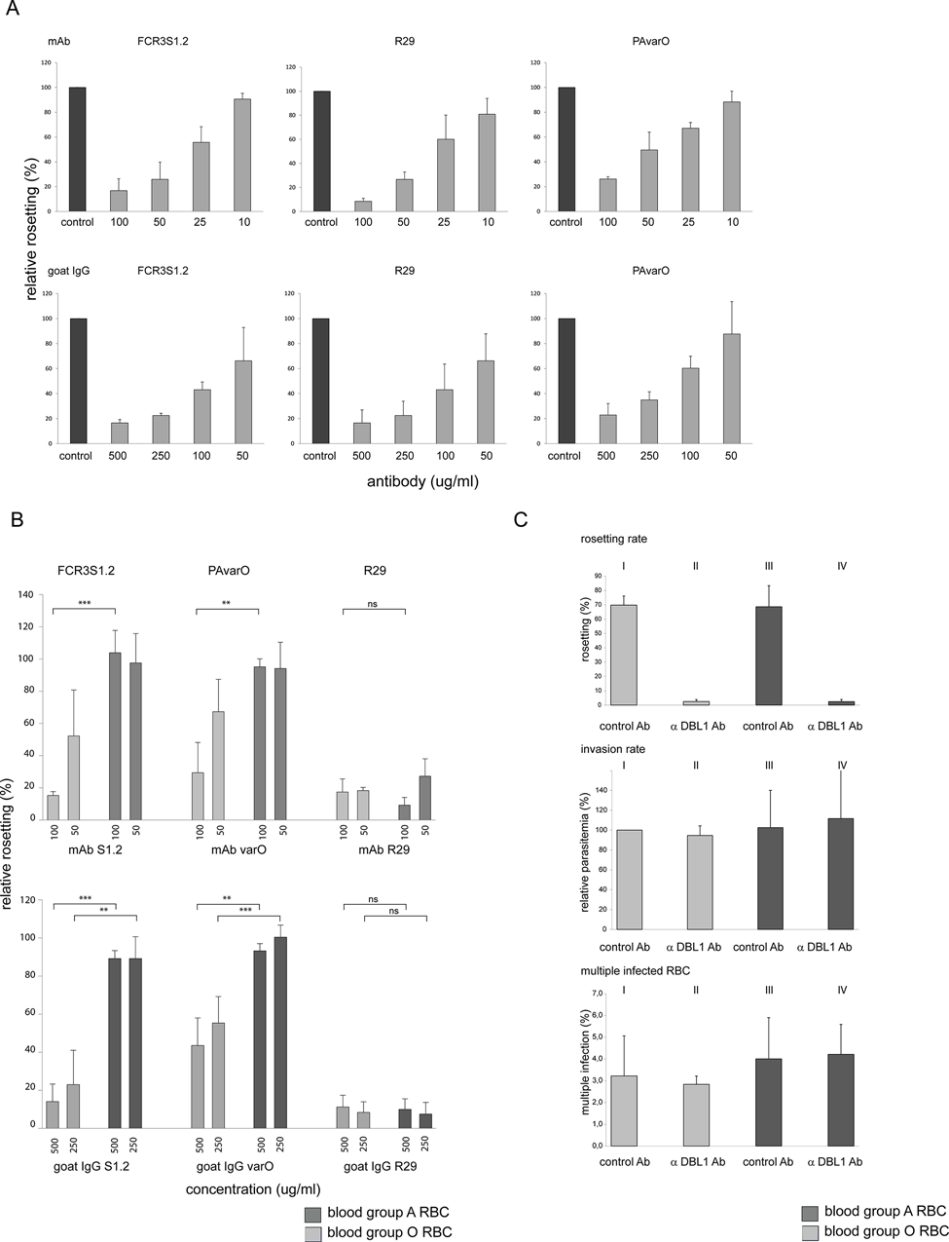
Rosette disruption with anti-DBL1α antibodies. **A.** Titration of anti-PfEMP1 antibodies: Prior to comparison of rosette disruption in blood group A versus O, antibodies were tested in different dilutions on the corresponding parasite in blood group O RBC for rosette disruption to verify a comparable affinity of the antibodies. Upper panel: monoclonal antibodies in concentrations of 100, 50, 25 and 10 μg/ml; lower panel: polyclonal goat IgG in concentrations of 500, 250, 100 and 50 μg/ml. Bars represent mean of 3 experiments plus SD. **B.** Disruption of rosettes by strain-specific anti-DBL1α antibodies: Parasites (FCR3S1.2, PAvarO, R29) to which strain-specific antibodies were available were assayed for rosette disruption in two different concentrations in parallel in blood group A and O. FCR3S1.2 and PAvarO rosettes of blood group A were resistant to anti-DBL1α antibodies, while rosettes were readily broken in blood group O. **Upper panel:** Rosette disruption with monoclonal anti-DBL1α antibodies at 100 and 50 μg/ml (FCR3S1.2 100 μg/ml p = 0.0004; PAvarO 100 μg/ml p = 0,0042; unpaired t test, two tailed). However, R29 showed sensitivity to anti-DBL1α antibodies in both blood groups (R29 100 μg/ml p = 0,2143; unpaired t test, two tailed) as previously shown [35]. Bars represent mean of 3 experiments plus SD. **Lower panel:** Rosette disruption with polyclonal goat IgG at 500 and 250 μg/ml (FCR3S1.2 500 μg/ml p = 0.0002; 250 μg/ml p = 0.0058; PAvarO 500 μg/ml p = 0.0046; 250 μg/ml p = 0.0003; unpaired t test, two tailed). However, R29 showed sensitivity to anti-DBL1α antibodies in both blood groups (R29 500 μg/ml p = 0.7997; 250 μg/ml p = 0.8611; unpaired t test, two tailed) as previously shown [35]. Bars represent mean of 3 experiments plus SD. Results of rosette disruption has partly been presented in [15,34]. **C.** long term *in vitro* growth: FCR3S1.2 pRBC were grown from ring to trophozoite stage in the presence of anti-DBL1α-IT_var60_ goat IgG (50 μg/ml) under 4 different conditions: I: blood group A RBC allowing rosetting, II: blood group A RBC inhibiting rosetting, III: blood group O RBC allowing rosetting, IV: blood group O RBC inhibiting rosetting. For all four conditions, rosetting rate, invasion rate and rate of multiple infected RBC was analyzed. No significant difference for these parameters could be observed for the four different conditions. Bars represent mean of 3 experiments plus SD.

To further analyze the preference and phenotype of rosettes grown in blood group A as compared to O RBC, three laboratory clones (FCR3S1.2, R29 and PAvarO) were propagated in parallel in blood group O or A to assay the sensitivity of rosettes for disruption by parasite strain-specific anti-PfEMP1-DBL1α mAb and polyclonal antibodies [30, 33]. 3D7S8.4 and the patient isolates were not included in this study since no strain-specific anti-PfEMP1-DBL1α antibodies were available for these parasites. Prior to the rosette disruption experiments, we tested the mAb and polyclonal goat IgG (pgIgG) to PfEMP1 [33] at different dilutions on pRBC grown in blood group O to verify comparable affinities of the antibodies (Fig 2A **).** From these experiments, concentrations of 50 and 100 μg/ml were chosen for the mAbs for subsequent experiments while 250 and 500 μg/ml were used of the pgIgG.

We found that group O-rosettes of FCR3S1.2 and PAvarO where sensitive to the antibodies (100 μg/ml 15% and 29% rosetting; 50 μg/ml 52% and 67% rosetting) while A-rosettes of the same two parasites were not (FCR3S1.2: 100 μg/ml 104% rosetting; 50 μg/ml 98% rosetting; PAvarO: 100 μg/ml 95% rosetting; 50 μg/ml 94% rosetting; FCR3S1.2 100 μg/ml p = 0.0004; PAvarO 100 μg/ml p = 0,0042; unpaired t test, two tailed). (Fig 2B, upper panel). In contrast, rosettes of the clone R29 were equally disrupted by strain-specific mAb anti-PfEMP1-DBL1α antibodies when propagated in blood group O or A RBC (blood group A: mAb 100 μg/ml 9% rosetting; mAb 50 μg/ml 27% rosetting; blood group O: mAb 100 μg/ml 17% rosetting; mAb 50 μg/ml 18% rosetting; 100 μg/ml; p = 0,2143; unpaired t test, two tailed) (Fig 2B, upper panel).

Similar results were obtained when polyclonal, strain specific anti-PfEMP1-DBL1α gpIgG were used for rosette-disruption of group O-rosettes of FCR3S1.2 or PAvarO (FCR3S1.2: 500 μg/ml 14% rosetting; 250 μg/ml 23% rosetting; PAvarO (500 μg/ml 44% rosetting; 250 μg/ml 55% rosetting) while A-rosettes of the same parasites were not sensitive to the pgIgG (FCR3S1.2: 500 μg/ml 89% rosetting; 250 μg/ml 89% rosetting; PAvarO: 500 μg/ml 93% rosetting; 250 μg/ml 100% rosetting; FCR3S1.2 500 μg/ml p = 0.0002, 250μg/ml p = 0.0058; PAvarO 500 μg/ml p = 0.0046, 250 μg/ml p = 0.0003; unpaired t test, two tailed), (Fig 2B, lower panel). In contrast, rosettes of the clone R29 were equally disrupted by strain-specific anti-DBL1α antibodies both when propagated in blood group O or A RBC (blood group A: 500 μg/ml 4% rosetting, 250 μg/ml 6% rosetting; blood group O: 500 μg/ml 11% rosetting, 250 μg/ml 8% rosetting; 500 μg/ml, p = 0.7997, 250 μg/ml, p = 0.8611; unpaired t test, two tailed) as previously shown by Ghumra et al. [34] (Fig 2B, lower panel).

Two of the three investigated parasite clones (FCR3S1.2, PAvarO) differ significantly in their sensitivity of rosettes towards anti-PfEMP1-DBL1α antibodies in blood group A versus O, while R29 showed equal sensitivity to the antibodies in the two blood groups.

### Rosetting does not give an advantage in multiplication or growth rate

To test the hypothesis whether the different types of rosettes lead to differences in invasion efficiency, we have analyzed the multiplication rates of the two laboratory parasite clones (FCR3S1.2, PAvarO) that form significantly larger rosettes in blood group A versus O and R29 that does not (Figs 1 and 2). Parallel cultures were set up with late trophozoite stage pRBC at the same parasitemia and were grown under shaking conditions with four RBC preparations of different ABO blood groups. Two were of blood group A with high- (A1) or low (A2) expression of the A blood group, one of group B and one of group O. Parasitemia was determined after the invasion process was complete after ≈ 24 hours. All three parasite clones showed a comparable multiplication rate in all tested blood preparations (multiplication rates: FCR3S1.2, group A1: 2.6, group A2: 2.6, group B: 3.1, group O: 2.8; PAvarO group A1: 3.1; group A2: 3.8, group B: 2.9, group O: 3.4; R29 group A1: 2.8; group A2: 3.3, group B: group 3.7, group O: 3.7). No statistically significant differences were observed (**S1** Fig) and the formation of larger rosettes in blood group A RBC for the parasite clones FCR3S1.2 and PAvarO were not in correlation with a more efficient invasion or growth rate under the tested *in vitro* conditions.

In a second set of experiments, the effect of rosette formation on multiplication and growth rate was compared within the same parasite clone propagated in the presence or absence of rosettes. FCR3S1.2 was grown in blood group A or O RBC respectively in the presence of pgIgG anti-PfEMP1-DBL1α-IT_var60_ or non-related pgIgG. This setting enabled us to compare rosetting- or non-rosetting FCR3S1.2 cultures for growth and invasion in blood groups A and O RBC at otherwise identical conditions where the parasites were gassed and shaken. Rosetting was documented every 48 h when the parasite reached trophozoite stage (28–30 hpi) for three consecutive 48 h asexual multiplication cycles (6 days). When the pgIgG anti-PfEMP1-DBL1α was added to the culture at ring stage the parasite did not form rosettes independently whether the RBC were of groups O or A (mean 3% RR, O RBC; mean 2%, RR A RBC). In contrast, if the parasites were grown in non-related pgIgG a RR of a mean of around 70% was seen both with O and A RBC (Fig 2C, upper panel). Different parameters of the cultures were studied during the experiment. Parasitemia was estimated every 48 h when the parasites had completed invasion into new RBC at 12–16 hpi. Analysis of the invasion rates revealed no significant differences in between the four different cultures (Fig 2C, middle panel). To further analyze if there was a growth advantage for pRBC in rosettes, the length of the asexual cycle and the stages of the parasites in the different cultures at the end of the 6 day period was monitored by microscopy. However, pRBC propagated at the four different culture conditions reached the same asexual stage at the same time point and it could not be concluded that rosette formation allows the parasite to complete its life cycle faster (data not shown). The number of multiple infected pRBC, parasitized by two or more parasites, was also examined but all four cultures displayed similar percentages of multiple-infected pRBC with no significant differences in the cultures grown in blood group A versus O RBC (mean multiple infection rate group O + pgIgG control 3.2%; group O + pgIgG anti-PfEMP1-DBL1-IT_var60_ 2.8%; group A + pgIgG control 4%; group A + pgIgG anti-PfEMP1-DBL1-IT_var60_ 4.2%; Fig 2C, lower panel). In general, the percentage of multiple infected pRBC increased with increasing parasitemia. In summary, no differences in growth, invasion or multiplication rates, multiple infected pRBC or length of the asexual multiplication-cycle were observed with any of the parasite clones studied, there were no correlations as to the ABO blood group used for the propagation of the parasites.

### Rosettes of blood group A impair antibody recognition of PfEMP1

**Fig 3:**
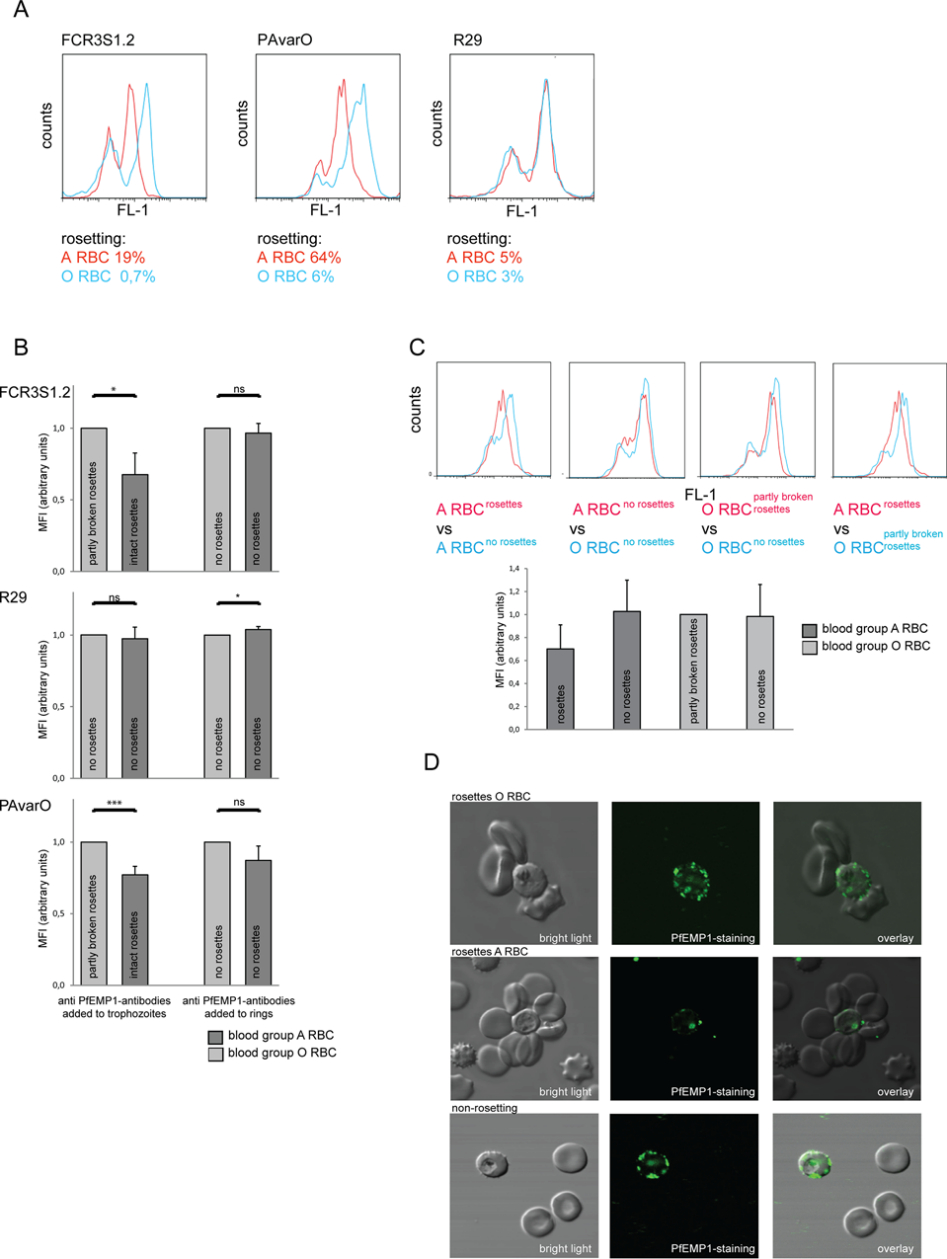
Accessibility of antibodies to surface expressed PfEMP1 in rosettes of blood group A versus O. Strain-specific anti-DBL1α goat IgG (10 μg/ml) was used to detect surface expressed PfEMP1 in the parasite clones FCR3S1.2, PAvarO and R29 comparing cultures grown in blood group A and O. **A:** FACS blot overlays: For FCR3S1.2 and PAvarO remaining rosettes could mainly be observed after the staining procedure in blood group A, while in R29 rosettes were extinct. The cultures of FCR3S1.2 and PAvarO with remaining rosettes in blood group A showed a reduced surface reactivity with anti-DBL1α antibodies as compared to blood group O where rosettes were removed with a strain-specific anti-DBL1α antibody. In contrast, no difference in staining intensity was seen in R29 as comparing the two blood groups, where rosetting was abolished under both conditions. FACS blots show a representative example of one experiment out of five. **B:** Mean fluorescence intensity of PfEMP1-staining on the pRBC surface in blood group A versus O in presence or absence of rosettes: Cultures in blood group O and A of FCR3S1.2, PAvarO and R29 were compared for their MFI for PfEMP1 staining. FCR3S1.2 and PAvarO showed a significant higher MFI in blood group O with partly broken rosettes as compared to blood group A with intact rosettes (FCR3S1.2 p = 0,0214; PAVarO p = 0,0005) which disappeared after abolishment of rosetting (FCR3S1.2 p = 0,4874; PAVarO p = 0,0686). R29 with rosettes sensitive to anti-DBL1α antibodies displayed no difference in cultures in blood group A versus O when antibodies are added to trophozoite stage (p = 0,5561) and an even higher recognition in blood group A when antibodies are added to ring stages (p = 0,03). MFI was adjusted to arbitrary units for each experiment; bars show the mean of three experiments plus SD. Unpaired t test, two tailed was used to identify p values. **C:** Accessibility of PfEMP1 on the pRBC surface in rosetting versus non-rosetting pRBC of FCR3S1.2: Upper panel: FCR3S1.2 pRBC were grown in blood group A and O under rosetting and non-rosetting conditions and accessibility of PfEMP1 on the pRBC surface was assayed with a strain-specific anti-DBL1α goat IgG (50 μg/ml). Formation of rosettes in blood group A reduces antibody accessibility of PfEMP1; even in blood group O staining is less intense when partially rosetting as compared to complete abolishment of rosettes. After removal of rosettes, both blood groups show a similar staining intensity. FACS blot shows a representative example of one experiment out of three of generation three. Lower panel: Average MFI for PfEMP1 staining of cultures of generation one, two and three as described above was compared for rosetting and non-rosetting conditions in blood group A and O, statistical analysis did not show any significant difference. **D:** Impaired accessibility of the pRBC surface for anti-PfEMP1 antibodies within a rosette as seen by indirect immunofluorescence: FCR3S1.2 pRBC were labelled with anti-DBL1α antibodies visualized in confocal microscopy using 10 μg/ml anti-DBL1α goat IgG and anti-goat-Alexa488. PfEMP1 expressed on the pRBC surface is labelled, but antibodies are hampered in accessing PfEMP1 by uninfected RBC bound to the pRBC surface. pRBC in smaller rosettes as in blood group O (upper panel) are more accessible as compared to pRBC in larger rosettes with more RBC as in blood group A (middle panel). Staining over the whole pRBC surface is seen for non-rosetting pRBC (lower panel).

The exposure of PfEMP1 at the pRBC surface was further analyzed with strain-specific anti-PfEMP1-DBL1α antibodies when pRBC were grown in blood group A or O. pRBC of parasites FCR3S1.2, PAvarO and R29 were assayed by flow cytometry in order to compare the amount of accessible PfEMP1 on the pRBC surface. In a first step, parasites grown for one generation in group A or O RBC were analyzed. Rosettes of parasites grown in blood group A that were resistant to anti-PfEMP1-DBL1α antibodies still revealed partial breakage of rosettes after the completed FACS staining-procedure likely due to the numerous washing steps, removal of serum components in combination with antibody incubation. Nevertheless, they retained a higher RR in blood group A vs. O (FCR3S1.2, 19% in BG A vs. 0.7% in BG O; PAvarO, 64% in BG A vs. 6% in blood group O). A correlated and more intense surface staining of the pRBC of these parasite clones was also observed in blood group O as compared to A (Fig 3A). In contrast, pRBC of R29 showed an equally low rosetting rate in both blood groups after staining (5% in A vs. 3% in BG O) and did not differ in surface staining for PfEMP1 in FACS (Fig 3A).

To quantify the difference in surface staining of PfEMP1 in relationship to the masking by RBC and to investigate if the blood group of the pRBC influences results, an experiment was designed where parasites FCR3S1.2, R29 or PAvarO were grown from ring to trophozoite stages in the presence of strain-specific pgIgG anti-PfEMP1-DBL1α and directly stained and studied in FACS. The experimental set-up results in non-rosetting cultures in both blood groups A or O that were compared to cultures where the antibodies were added to rosetting RBC infected by trophozoite-stage parasites. The mean fluorescence intensity (MFI) of the pRBC was measured and the MFI of parasites in blood group O was set as arbitrary unit 1 and MFI of blood group A related to it. Experiments were carried out three times, O RBCs were obtained from three different mixtures of two donors each (six donors total), A RBCs were from one donor in each experiment (three donors total). Addition of the anti-PfEMP1-DBL1α antibodies to trophozoite stage-pRBC displayed a MFI in FCR3S1.2 that was significantly decreased with the presence of rosettes in blood group A as compared to a partially rosetting culture of blood group O (p = 0,0214) (Fig 3B). However, when antibodies were added to rings and grown to trophozoites that were non-rosetting, the staining intensity was similar for both blood groups (p = 0,4874). Correspondingly, a highly significant difference for MFI comparing rosetting cultures of blood group A and partly rosetting of blood group O was seen for PAvarO (p = 0,0005) that disappeared with the growth with the pgIgG that prevents rosettes to form in culture (p = 0,0686). For R29, a parasite that loses rosettes in the presence of anti-PfEMP1-DBL1α antibodies in both blood groups, no difference in MFI could be observed when antibodies were added to trophozoite stages (p = 0,5561), growing R29 in the presence of antibody from ring to trophozoite stages however displayed a MFI higher in blood group A as compared to O (p = 0,03) (Fig 3B).

In further experiments we grew FCR3S1.2 parasites in blood group A or O RBC for three generations in the presence of strain specific anti-PfEMP1-DBL1α- or control pgIgG in four different set-ups: 1) FCR3S1.2 in blood group A, rosetting in the presence of control pgIgG, 2) FCR3S1.2 in blood group A, non-rosetting in the presence of strain-specific pgIgG anti-PfEMP1DBL1α-IT_var60_, 3) FCR3S1.2 in blood group O, rosetting in the presence of control pgIgG, and 4) FCR3S1.2 in blood group O RBC, non-rosetting in the presence of pgIgG anti-PfEMP1DBL1α-IT_var60_. The accessibility of PfEMP1 for strain-specific anti-DBL1α antibodies was studied after one, two and three generations. Staining intensity was found to be independent of the blood group of the invaded RBC as seen when the pRBC were grown all three cycles with specific pgIgG since the pRBC showed similar surface staining in blood group A and O RBC. In contrast, in cultures grown with the control pgIgG the formation of rosettes was found to protect the surface expressed PfEMP1 from being recognized by specific antibodies. As in previous experiments, formation of rosettes in A RBC displayed the strong protection against antibody recognition, as staining intensity of rosetting pRBC in blood group A was lowest since rosettes withstand manipulation during the staining procedure better as compared to blood group O rosettes. This is presumably due to the stronger and tighter rosettes formed in blood group A and the larger number of RBC bound per pRBC (Fig 3C **,** results of generation three upper panel, MFI: mean of three generations lower panel).

To confirm the data we also visualized the surface staining of FCR3S1.2 pRBC involved in rosettes of blood group O or A in confocal microscopy; staining of live pRBC with strain specific pgIgG showed stronger labelling of the pRBC in rosettes of blood group O as compared to blood group A labelling over the whole pRBC-surface was observed with pRBC not incorporated in a rosette (Fig 3D).

The presence of antibody-resistant blood group A rosettes leads to a diminished reactivity, lower recognition of the pRBC by anti-PfEMP1-DBL1α antibodies. Importantly, removal of rosettes shows that PfEMP1 is indeed expressed at similar levels at the surface of blood group A and O pRBC.

### Non-immune IgM is bound to the pRBC surface in blood group A and O in FCR3S1.2

**Fig 4:**
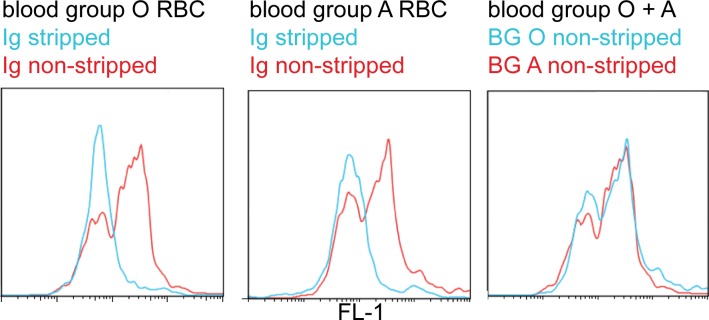
Presence of non-immune IgM on the pRBC surface in blood group A versus O. FCR3S1.2 pRBC grown in blood group A and O were assayed for the presence of non-immune human Ig on the pRBC surface with a FITC-labeled α-human Ig antibody. Since pRBC of FCR3S1.2 are known to bind only non-immune IgM, but not IgG, the graph displays IgM binding. Control pRBC were stripped with Sodiumcitrate to remove Ig from the cell surface and were compared to untreated pRBC. pRBC in both blood groups bind Ig to the same extent. FACS blot shows a representative example of one experiment out of three.

Binding of non-immune immunoglobulins has an important role in the formation of rosettes in parasite clones such as FCR3S1.2 [35]. Therefore, the capacity to bind immunoglobulins to the pRBC surface was also investigated in parasites grown in blood group A as compared to O. pRBC were either taken straight from the culture, or stripped twice of serum-proteins bound to the cell-surface with sodiumcitrate [36], and then stained with a goat α-human-Ig antibody coupled to FITC. A typical punctate staining was seen on pRBC of the untreated cultures while sodium citrate treatment efficiently removed all Ig bound to the pRBC surface in both blood groups, as expected. No difference in amount of non-immune Ig present at the pRBC surface could be observed in between the pRBC of the different ABO blood groups treated in a similar manner in FACS (Fig 4). This excludes that differences in binding of IgM to the pRBC surface in blood group A or O contributes to the different rosetting features in the two blood groups, which was also emphasized by a similar levels of sensitivity of rosettes to sodium citrate treatment (data not shown).

## Discussion

The ability of the parasite to form rosettes with uninfected host RBC taken together with the patient’s ABO blood group has been suggested to be of fundamental importance for the outcome of a malaria infection [7,8,14,37,38]. Rosette-formation causes obstruction of the blood flow, tissue ischemia and cell death [39,40] and is possibly one of the major factors influencing the outcome of malaria in patients of different blood groups of the ABO system [11]. The size of rosettes vary with the ABO-blood group such that rosettes formed by RBC of blood group A, B or AB are larger and are held together with stronger forces than rosettes formed by group O RBC [13,15]. The importance of these rosetting parameters for the disease outcome have also been suggested to be at hand for individuals with hemoglobin variants such as thalassaemia and sickle-hemoglobin, which show a reduced risk of severe disease albeit the RBC phenotypes are not protective against infection per se. Since reduced binding of pRBC to host cells correlates with the altered display of PfEM, 1 it has also been suggested that small uninfected red cells or sickle-RBC bind less to pRBC thereby giving rise to fewer, weakly adhesive and/or smaller rosettes [41–45]. In a similar way, individuals of different blood groups of the ABO system do not vary in their susceptibility to malaria infection as such [46], but differ strikingly in their predispositions to come down with severe malaria. As shown here, rosettes formed in blood group A RBC are larger in a subgroup of parasite strains, both among laboratory strains and patient isolates, and rosettes are tight and difficult to disrupt by specific antibodies. The ability to rosette is consequently considered a virulence factor in *P*. *falciparum* [23,29,47–49], additionally, rosetting in a host of blood group A is likely a setting where the already rosetting parasite is comparatively more virulent than in a patient of blood group O.

Herein we show that rosettes formed in blood group A RBC make the pRBC less accessible to anti-PfEMP1antibodies and more resistant to rosette disruption. Rosetting in blood group O is based on the interaction between the DBL1α domain of the parasite derived ligand PfEMP1 and receptors on the RBC surface and antibodies targeting this domain readily break the interaction with blood group O but not in blood group A RBC [29,30,50,51]. In a detailed analysis it has been demonstrated that antibodies to epitopes of a minimal region, including basic amino acids in subdomain 3 of the DBL1α-PfEMP1, are sufficient to block rosette formation with blood group O RBC [51,52] although blood group O rosetting sometimes also relies on the RIFINs for binding. For example, RIFIN is expressed at the surface of pRBC of parasite clone FCR3S1.2, where RIFIN-binding to sialic acid on glycophorin A further strengthens the interaction of the pRBC with the RBC of blood group O [15]. In contrast, rosetting in blood group A relies not only on the interaction with receptors present on any RBC as heparan sulfate, sialic acid and IgM, but in addition on binding to the blood group A antigen mediated by members of the RIFIN family as recently identified [15]. The role of PfEMP1-DBL1α binding to blood group A has also been suggested [53] but could not be confirmed by the experiments herein.

Disruption of rosettes in blood group A is complex to achieve as it requires acquisition of antibodies to various critical epitopes. Therefore, only affinity purified antibodies to RIFIN affect blood group A rosetting, but do not break rosettes of blood group O RBC [15], while anti-PfEMP1 antibodies break rosettes of blood group O but not A when added to already formed rosettes in FCR3S1.2. However, PfEMP1-antibodies can prevent the formation of rosettes if present while the parasites mature from ring- into trophozoite stage. Due to the large size of PfEMP1, about seven times that of RIFIN (40,000 Da), antibodies to PfEMP1 presumably hinder the RIFIN to bind RBC sterically when present prior to the rosette formation. The need for a more complex composition of protective antibodies to inhibit these interactions is possibly one explanation for the underlying mechanism of the increased virulence of parasites preferring blood group A for rosetting.

Group A RBC may also allow the merozoite to evade certain, but maybe not all, anti-merozoite immune responses in highly rosetting parasites. Further, the formation of rosettes by four species of the human-infecting *Plasmodiae* [54] suggests that rosetting is important for a successful infection. Previous studies have both favored and rebutted the hypothesis that merozoites released from RBC bound in a rosette more easily complete the invasion process [20,21,25,55–57], an advantage that could not be observed in the parasite clones investigated herein, as larger and tighter rosettes did not result in higher invasion rates. However, during *in vitro* growth of *P*. *falciparum* the merozoite is not exposed to any immunological pressure and the advantage of invasion into a RBC bound to the endothelium and in a rosette avoiding exposure to the immune system is partly absent in an *in vitro* system. Still, *P*. *vivax* has recently been found to preferably form rosettes not with reticulocytes but mature RBC, which cannot be invaded by the *P*. *vivax* parasite. The same study demonstrated that gametocytes in *P*. *vivax* as well as *P*. *falciparum* also have the ability to form rosettes. These observations point at a function of rosetting different from facilitated invasion of the merozoite [58,59]. The findings herein suggest that rosetting functions as a “cloaking device” protecting the pRBC from effector mechanisms of the immune system as suggested by earlier studies [19,27]. This line of thought has been suggested for the rosette forming organism *Borrelia crocidurae* for which an increased bacteria load and a reduced production of specific antibodies can be observed during infection [60].

We here show that PfEMP1-specific antibodies are less prone to bind to the surface exposed PfEMP1 and less able to disrupt rosettes when parasites are grown in group A versus group O RBC since the tighter rosettes formed in blood group A RBC hinder recognition. Thus, rosette formation gives the parasite an advantage by blocking the pRBC from antibody-binding and subsequent clearance by host phagocytic cells [61] and increased phagocytosis of parasitized O as compared to A RBC has been demonstrated [62]. The survival advantage for the parasite in A RBC may therfore lead to higher parasitemia and compound clearance of the infection. This phenomenon is most distinct when parasites face blood group A erythrocytes providing a possible explanation why the ABO blood group system is so important for the outcome of severe malaria.

## Material and Methods

### Ethics statement

The study was approved by Karolinska Institute’s Regional Ethical Review Board (permission 420 03/095) and the Uganda National Council for Science and Technology (permission MV717). Written informed consent was obtained from the parents or guardians of the patients.

### Parasite cultures

*P*. *falciparum* laboratory clones were cultivated according to standard methods [63]. Patient isolates were collected in Kampala, Uganda [32]; establishment of cultures was carried out as described before [21]. All parasite cultures were kept under shaking conditions during all experiments.

The rosetting phenotype of FCR3S1.2 and 3D7S8.4 was maintained by enrichment over a Ficoll-gradient [63], while enrichment on mAbs was carried out as described for R29 and PAvarO [30].

Cultures were long-term propagated in O erythrocytes, for experiments, parasites were sub-cultured in A erythrocytes and assayed after one generation, except in experiments where indicated for longer growth.

Rosetting and non-rosetting cultures of the parasite clones in both blood groups were established by adding 50 μg/ml anti-DBL1α goat IgG or normal goat IgG (negative control) into the culture medium and were grown in presence of antibodies from ring stage onwards.

Rosette size was determined by counting the number of bound erythrocytes in 100 rosettes by staining the parasite culture with Acridine Orange and microscopic investigation. Similar, multiple infections were investigated by staining the parasite culture with Acridine Orange and microscopic investigation of 300 pRBC.

Parasitemia was determined by staining the parasite culture with 2.5μg/ml Ethidium Bromide for 5 min at RT. The cell acquisition was done using flow cytometry (FACSCalibur, BD Bioscience) where 100 000 total events were counted.

### Blood group preference in formation of rosettes

pRBC (in blood group O) older than 24h p.i. were enriched by MACS as described earlier ([63]) and resuspended in malaria complete medium with 10% human serum (MCM S+). Normal RBC of blood group A and O were each labelled with PKH of one out of two different colours (Sigma, MINI67-1KT, MINI26-1KT). Staining was carried out using PKH67 for green and PKH26 for red labelling. 1 μl of RBC was washed twice with RPMI and mixed with 125 μl Dilutent C, thereafter a mixture of 125 μl Dilutent C plus 0.5 μl PKH 67 or 26 was added and incubated for 5 min at RT; 250 μl of PBS/FCS 3% were added and the cells washed twice with RPMI. RBC were resuspended in (MCM S+) to 5% hematocrit; RBC of both blood groups labelled in two different colours were mixed to same percentage and purified pRBC were added to a final parasitemia of 10%; formation of agglutinates of MACS enriched pRBC was mechanically broken by passage through a needle directly before addition to the experiment. Formation of rosettes was allowed for 60 min at RT and the percentage of blood group A or O RBC was determined by microscopic investigation of 100 individual rosettes.

### Blood group preference in invasion

pRBC older than 30h p.i. were enriched by MACS as described earlier [63] and resuspended in 100 μl of malaria complete medium with 10% human serum (MCM S+) and RBC of blood group A1, A2, B or O to a haematocrit of 5% and a parasitemia of 0.5% in two setups of triplicates in a 96 well plate. One set was immediately stained with a final concentration of 2.5μg/ml of ethidium bromide for 5 min at RT and analysed by flow cytometry to define the starting parasitemia of the assay. 200.000 cells were investigated with a FACSCalibur, BD Bioscience, http://www.bd.com), the analysis was performed using the software FlowJo. The second set of triplicates was analysed in the same way after the parasites had finished the invasion process and developed into late rings/early trophozoites.

### Rosette disruption assay

Strain-specific mouse monoclonal antibodies [51,53] towards the DBL1α domain of PfEMP1 or strain-specific goat IgG [33,51] were tested for disruption of rosettes of the different parasite clones as described [63]. Concentrations of 50 and 100 μg/ml of monoclonal antibodies as well as an unrelated control mAb; concentrations of 500 and 250 μg/ml of goat IgG as well as an non immune goat IgG as control were used.

### Analysis of surface expression of PfEMP1 by flow cytometry

Trophozoite-infected RBC in blood group A or O RBC (between 24 and 34 h p.i.) were incubated with a strain-specific goat IgG (10 μg/ml, [51,53] in PBS/FCS (2%) for 60 min at RT, followed by three washes with PBS/FCS. Afterwards, a 30 min incubation with anti-goat IgG second antibody coupled to ALEXA488 (Molecular Probes, dilution 1:200) was performed at RT. For nuclear staining Hoechst33342 (Invitrogen, final dilution 1:8000) was added together with the secondary antibody or ethidium bromide was added at final concentration of 2.5μg/ml for 5 min at RT. The pRBC were washed three times and resuspended in PBS/FCS. The cell acquisition was done using flow cytometry (FACSCalibur or FACSVerse; BD Bioscience) where 5000 pRBC were counted. The analysis was performed using the software FlowJo.

Non-immune human Ig bound to the pRBC suface was detected as above using an anti-human Ig FITC-coupled antibody (DAKO, F0200, dilution 1:100). As a negative control a monoclonal antibody generated in mouse against a non-related bacterial protein or normal goat IgG was used.

### Confocal microscopy

Live FCR3S1.2 trophozoite-infected RBC in blood group A or O RBC (between 24 and 34 h p.i.) were incubated with polyclonal goat IgG (10 μg/ml, strain-specific anti-DBL1α [51,53] in PBS/FCS (2%) for 60 min at RT, followed by three washes with PBS/FCS. Afterwards, a 30 min incubation with anti-goat IgG second antibody coupled to ALEXA488 (Molecular Probes, dilution 1:200) was performed at RT. For staining of pRBC not incorporated in a rosette, mechanical breakage of rosettes by passage through a needle was carried out prior to the staining procedure. Confocal images were captured with an Olympus Fluoview FV1000 (Japan) with a 473nm solid state laser, UPlanSApo 60x lens and a 6x digital zoom. Images were analysed with Olympus Fluoview version 4.0.

### Statistical analysis

Statistical analysis was carried out using unpaired t test, two tailed to analyze rosette size in blood group A versus O (Fig 1A); unpaired t test, two tailed p value to analyze the preference of incorporation of RBC of blood group A versus O in rosettes (Fig 1B); unpaired t test, two tailed to compare the inhibitory effect of anti-DBL1α antibodies on preformed rosettes comparing blood group A versus O (Fig 2B) and unpaired t test, two tailed to compare the recognition of surface expressed PfEMP1 expressed as mean fluorescence intensity in blood group O versus A (Fig 3 B).

## Supporting Information

S1 Fig Multiplication rate in different ABO blood groups. Parasite clones FCR3S1.2, PAvarO and R29 were grown in parallel in four different blood groups (A1, A2, B, O). The multiplication rate for each of the three parasite clones did not show any statistically significant difference in the different ABO blood groups. Bars represent mean of 3 experiments plus SD.
(TIF)
