# Correction: Evasion of Immunity to *Plasmodium falciparum*: Rosettes of Blood Group A Impair Recognition of PfEMP1

**DOI:** 10.1371/journal.pone.0149765

**Published:** 2016-02-12

**Authors:** Kirsten Moll, Mia Palmkvist, Junhong Ch'ng, Mpungu Steven Kiwuwa, Mats Wahlgren

The following information is missing from the Funding section: This study was supported by the Swedish Strategic Foundation, the EU Sixth- and Seventh-Framework Programs (MEST-CT-2004-8475 and EviMalar Network of Excellence), and the Swedish Research Council (VR/2012-2014/521-2011-3377).
