# Supplementary figures and images for: Evasion of Immunity to Plasmodium falciparum: Rosettes of Blood Group A Impair Recognition of PfEMP1
Source: PLoS One. 2015 Dec 29;10(12):e0145120. doi: 10.1371/journal.pone.0145120 (PMC4694710; doi:10.1371/journal.pone.0145120)

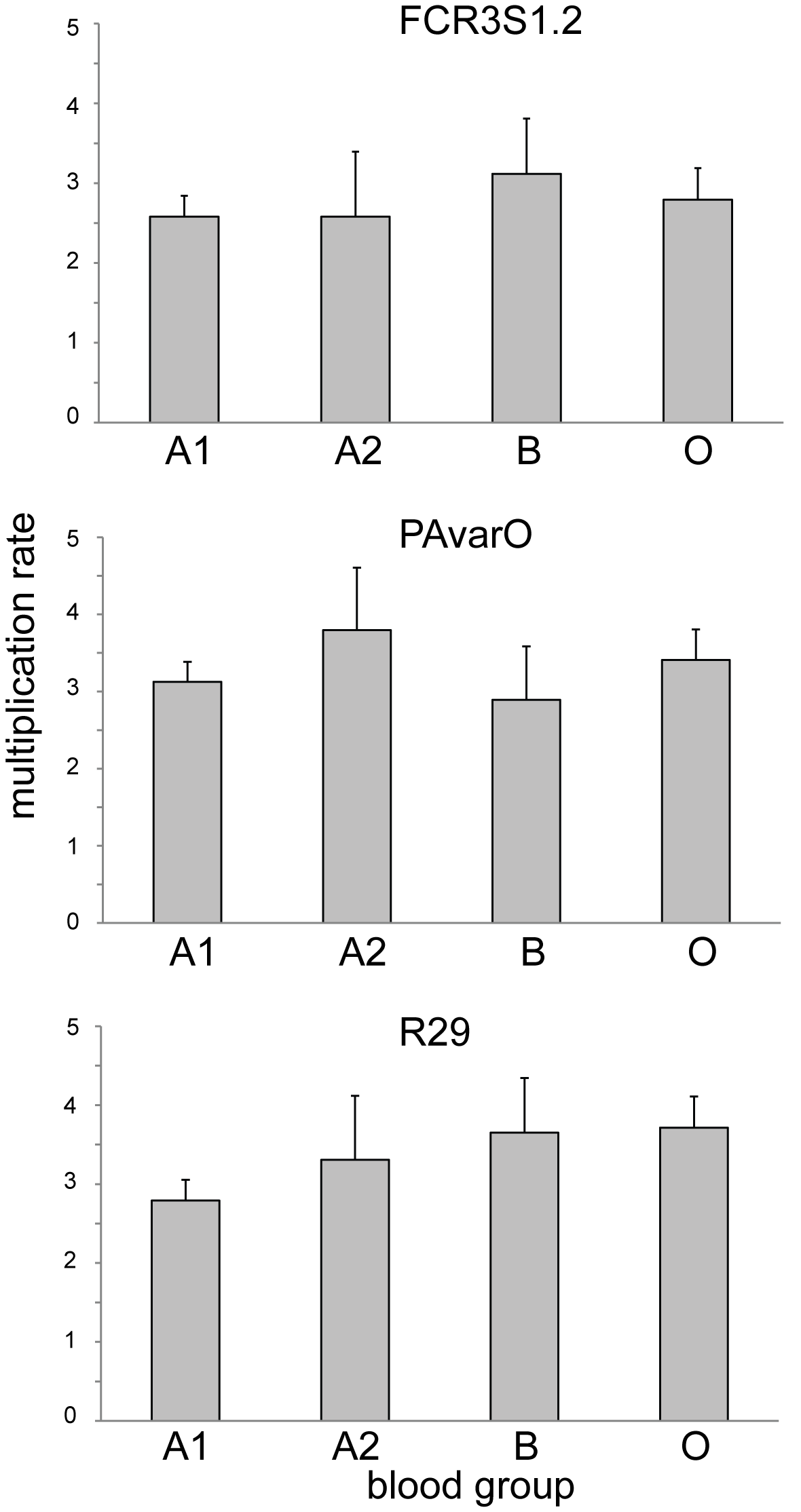

Supplement: S1 Fig — Parasite clones FCR3S1.2, PAvarO and R29 were grown in parallel in four different blood groups (A1, A2, B, O). The multiplication rate for each of the three parasite clones did not show any statistically significant difference in the different ABO blood groups. Bars represent mean of 3 experiments plus SD. (TIF) [file pone.0145120.s001.tif]
